# Baking Meats

**Published:** 1879-10

**Authors:** 


### RECIPES.

### BAKING MEATS.

In roasting or baking meats or poultry no water should be used; it greatly impairs their flavor. Instead of water, put a little lard or dripping in the pan. Have the oven thoroughly hot when the meats are put in, so that a crisp may be formed at once, which will retain the juices. If the meat is likely to burn, lay over it a piece of thin greased paper.

